# Closed-loop movement-paired transcutaneous auricular vagus nerve stimulation for upper-limb rehabilitation: a feasibility study

**DOI:** 10.1186/s12984-026-02021-7

**Published:** 2026-05-20

**Authors:** Clément Lhoste, Max Quast, Andrea Ronco, Abigail Vogel, Chris Easthope Awai, Meret Branscheidt, Olivier Lambercy, Paulius Viskaitis, Dane C Donegan

**Affiliations:** 1 Rehabilitation Engineering Laboratory, ETH Zurich, Zurich, Switzerland; 2 Department of Health Sciences and Technology, ETH Zurich, Zurich, Switzerland; 3 Data Analytics and Rehabilitation Technology (DART), Lake Lucerne Institute (LLUI), Vitznau, Switzerland; 4 cereneo, Center for Research and Neurorehabilitation, Weggis, Switzerland; 5 Neuroscience Center Zurich (ZNZ), University of Zurich, Federal Institute of Technology Zurich, University and Balgrist Hospital Zurich, Zurich, Switzerland; 6 Future Health Technologies, Singapore-ETH Centre, Campus for Research Excellence and Technological Enterprise (CREATE), Singapore, Singapore

## Abstract

Vagus nerve stimulation (VNS) paired with physical rehabilitation has shown promise for improving upper-limb function after neurological injury. In this study, we introduce SmartVNS, a wearable system that automatically delivers movement-paired transcutaneous auricular VNS using a wrist-worn intertial measurement unit (IMU) for accurate functional movement detection. We evaluated feasibility, usability and performance of the SmartVNS system in a four-week, 20-session clinical feasibility study involving nine individuals with stroke or spinal cord injury during conventional therapy. Despite a wide range of baseline upper-limb function in patients (ARAT scores 0-52), the system adapted to individual abilities and demonstrated a consistent stimulation rate between sessions and patients, averaging 14.7 stimulations/min with a low variability (SD = 3.4 stimulations/min; n = 9). The IMU-based movement detection algorithm achieved stimulation precision comparable to therapist manual triggering (precision as mean ± SEM: SmartVNS: 76.3 ± 3.1%, therapists: 82.4 ± 2.6%, video analysis used a ground truth), while delivering stimulation in double the number of movements (sensitivity as mean ± SEM: SmartVNS: 50.4 ± 5.0%, therapists: 23.4 ± 4.7%, video analysis used a ground truth). The system demonstrated high usability (patients and therapists rated UMUX: 85 ± 12%) and was well-tolerated by participants, with patients able to self-apply the device. Exploratory clinical outcomes showed consistent functional improvements across participants. These findings support the feasibility and usability of SmartVNS as a scalable neuromodulation platform to support neurorehabilitation.

**Supplementary Information:**

The online version contains supplementary material available at 10.1186/s12984-026-02021-7.

## Introduction

Neurological injuries, including stroke and spinal cord injury (SCI), are among the leading causes of disability worldwide [1]. A particularly common consequence of these conditions is upper-limb impairment, with persistent deficits affecting around 50% of stroke survivors even six months after [2]. To address this, movement therapy focuses on repeated practice of motor tasks, with recommendations underscoring the importance of high-dose training in promoting recovery [3–5]. However, as increasing therapy dose is challenging and resource-demanding, emerging strategies are being developed in parallel to enhance the mechanisms of conventional therapy. Among these, neuromodulation, which acts directly on the brain and its injured pathways, shows strong potential to enhance neuroplasticity and facilitate recovery beyond physical therapy alone [6–9].

As a promising neuromodulation approach, vagus nerve stimulation (VNS) has been shown to enhance upper-limb motor recovery. When paired with movement during upper-limb rehabilitation for stroke [10] and SCI [11], VNS further improves recovery compared to sham stimulation. This pairing of VNS during movement leverages activity-dependent hebbian and long-term potentiation (LTP) plasticity mechanisms—strengthening the synapses of task-relevant neural pathways—along with increased corticomotor drive, both essential features for recovery [12]. Despite these promising results, the widespread use of VNS faces significant accessibility barriers due to the requirement for surgical implantation. Even when accessible, this procedure is expensive and associated with possible severe adverse events [13, 14]. Furthermore, many patients are unwilling or ineligible to undergo surgery, especially in the acute/subacute phase of the injury [15].

Non-invasive transcutaneous auricular VNS (taVNS), targets the auricular branch of the vagus nerve (ABVN) via electrodes placed on the external ear, therefore offering a compelling alternative [6, 13, 16]. taVNS has been shown to engage central neuromodulatory centers, such as the locus coeruleus and ventral tegmental area, in a manner comparable to invasive VNS [17–19]. Furthermore, movement-paired stimulation has been demonstrated to increase cortical sensorimotor activity and corticospinal excitation on rapid timescales [20]. Building upon this, preliminary studies have demonstrated excellent safety of taVNS and suggest promising motor improvements when stimulation is paired with movement [21–25], with clinical effect sizes similar to invasive VNS [13, 21]. However, the clinical usability of movement-paired taVNS remains limited in its current form. Most implementations rely on external manual activation by therapists or complex hardware setups that prevent patients from independently initiating stimulation and impact usability [21]. These approaches introduce variability in stimulation delivery [26] and typically rely on supervised use, especially for individuals with severe motor impairments. This makes them impractical solutions for democratizing taVNS. To address these barriers, motion sensors such as inertial measurement units (IMU) could be used to automate stimulation based on movement detection, improving both the usability and consistency of taVNS delivery [11, 27].

This work introduces SmartVNS, a novel all-in-one system to automatically deliver movement-paired taVNS. SmartVNS is specifically engineered for high usability and autonomy in neurologically injured populations, aiming for a seamless integration into existing motor rehabilitation workflows. The system leverages a wrist-worn IMU combined with an adaptive algorithm to detect functional upper-limb movements in real time. It then delivers stimulation to the ABVN, without manual intervention, via a wearable custom-built stimulator connected to an optimized electrode. We hypothesized that the SmartVNS system would demonstrate high patient usability and clinical acceptance, and that its wrist-worn IMU sensor could enable automated and precise detection of functional movements during standard motor therapy. To evaluate our approach, nine individuals with stroke or SCI participated in daily SmartVNS-enhanced training over a four-week period. We assessed system usability, as well as movement detection, stimulation performance, and changes in upper-limb motor function before and after the intervention.

## Methods

### SmartVNS system and its components

**Fig. 1:**
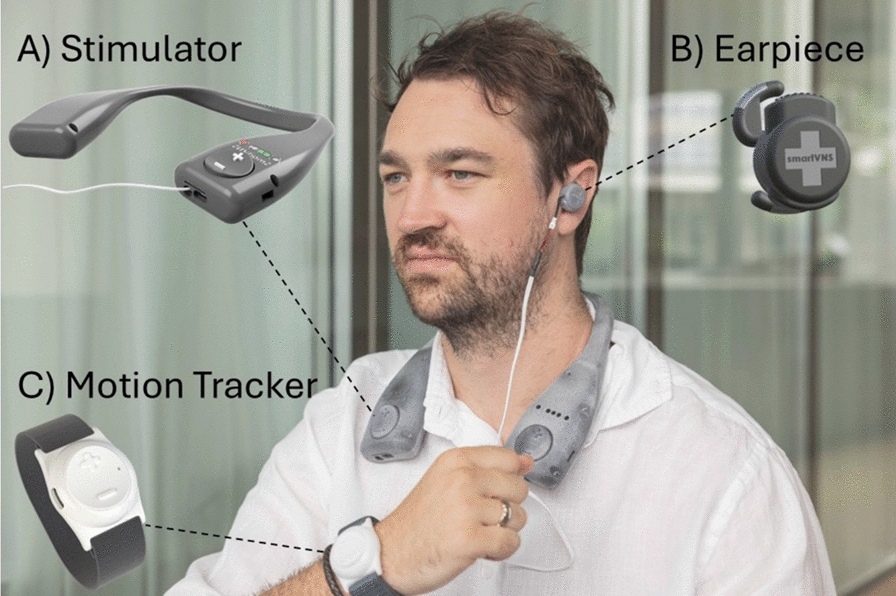
Overview of the SmartVNS system. (A) Stimulator unit: Enclosed in a horseshoe-shaped housing worn around the neck, the stimulator contains two rigid compartments that house the electronics (including plus and minus buttons to calibrate stimulation intensity, LEDs informing on system status), connected by a flexible segment for one-handed placement and comfortable wear. (B) Earpiece: Inserted into the ear contralateral to the trained limb, the earpiece includes a spring-loaded mechanism for easy insertion and stable skin contact. Three size variants were developed to accommodate different ear anatomies. (C) Motion tracker: A wearable sensor (IMU) used to monitor wrist movements and provide input for stimulation control

**Fig. 2:**
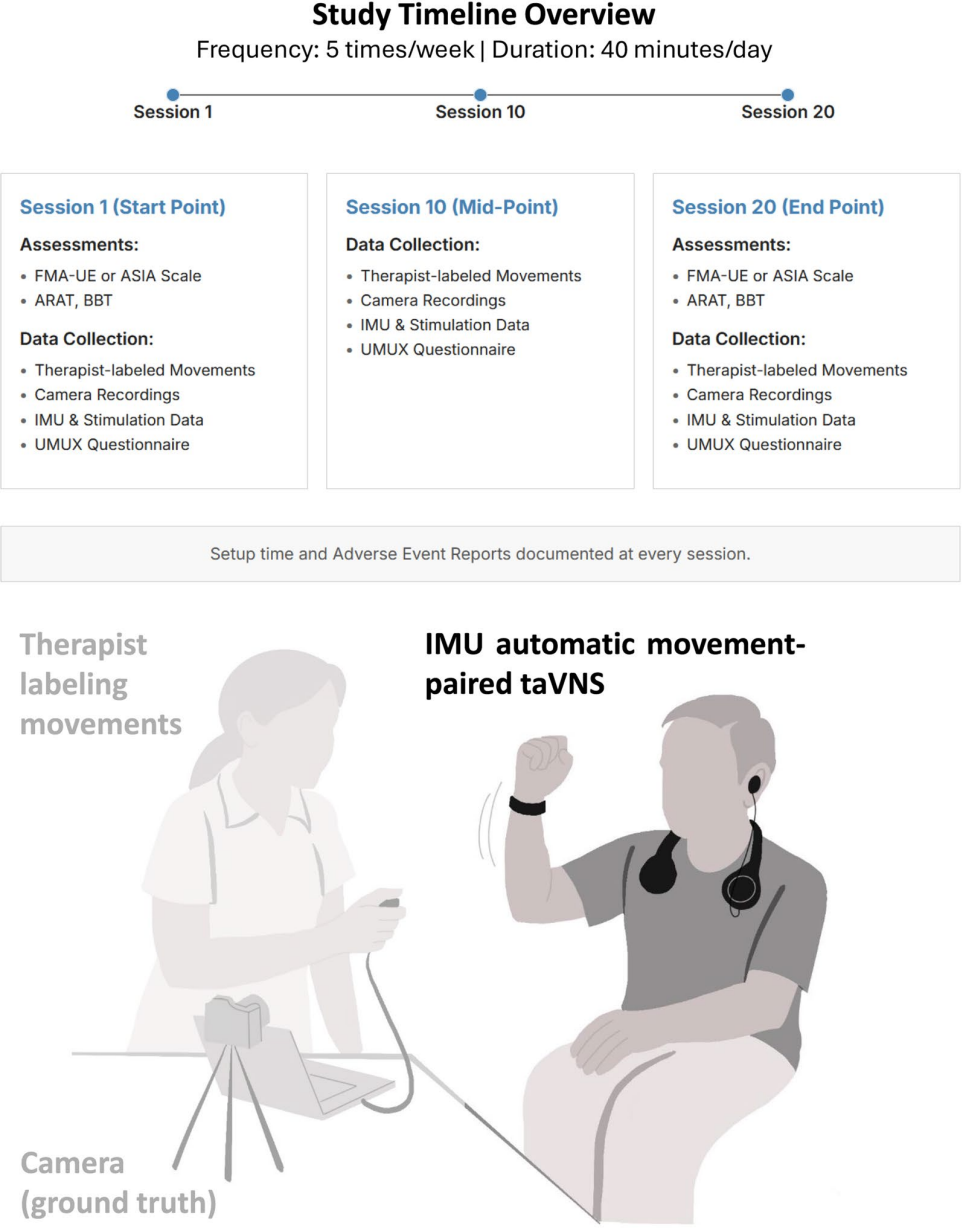
Overview of the 4-week intervention protocol. The intervention consisted of 20 sessions of 40 min of standard upper-limb therapy combined with the SmartVNS device. Key feasibility and usability assessments were performed at session 1 (baseline), session 10 (midpoint), and session 20 (post-intervention). Clinical scores were collected before and after the intervention

**Fig. 3:**
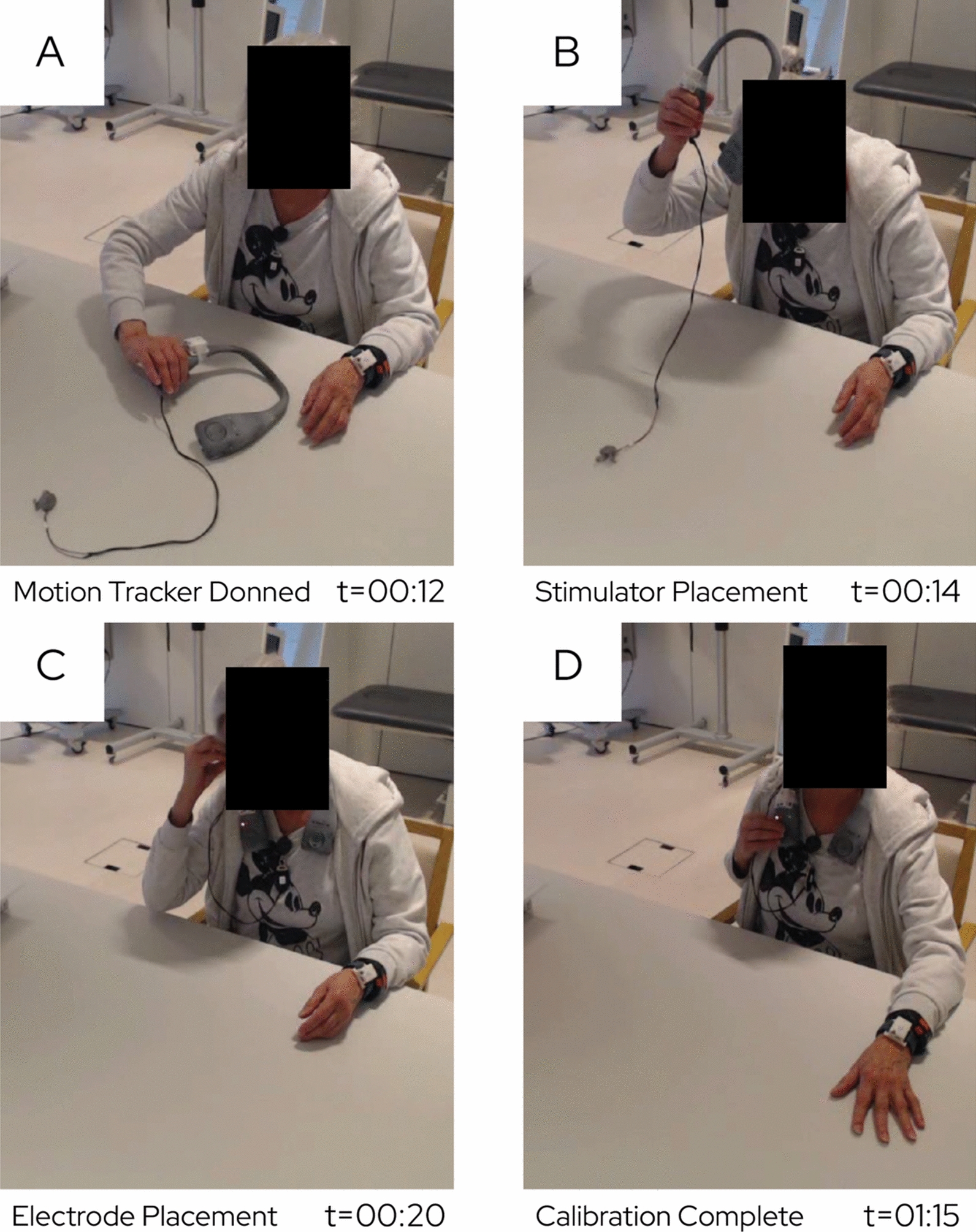
A patient with stroke independently placing the SmartVNS device using only the non-paretic hand. Time (mm:ss) indicates elapsed time from the start of the setup procedure. **A** Start of stimulation setup (motion tracker already donned, 12 s, not shown). **B** Placement of stimulator unit around the neck. **C** Placement of electrode in the ear. **D** Calibration of stimulation intensity

We developed in-house a closed-loop movement-paired taVNS system, referred to as SmartVNS. This device comprises three main components: (1) a stimulator unit worn around the neck that can deliver controlled electrical stimulation (current: 0–$5000 \mu A$, frequency: up to 10 kHz, biphasic pulse width: min $50 \mu$s, Fig. 1A), (2) an earpiece with a shape optimized to precisely position the stimulation electrodes to target the auricular branch of the vagus nerve (Fig. 1B), and (3) a wrist-worn motion tracker incorporating a 9-axis inertial measurement unit (IMU) for movement detection (Fig. 1C). Additionally, a laptop, communicating via bluetooth with stimulator and motion tracker units, is used for data processing, acquisition, and visualization.

The system was specifically designed for use by neurological patients, particularly users with hemiplegia following a stroke. Each component was designed for one-handed donning using the patient’s less affected arm. The IMU was worn on the affected arm, the stimulator unit around the neck and the earpiece inserted into the ear contralateral to the impaired limb [28]. The stimulator unit consists of a horseshoe-shaped housing with two rigid compartments for electronics connected by a flexible segment that facilitates simple positioning and adapts to user movements. Stimulation intensity can be adjusted via accessible plus and minus control buttons (steps of 100$\mu$A). LEDs inform of the state of the device (e.g., power on, stimulating, etc.). The earpiece, connected with a cable to the stimulator unit, incorporates a spring-loaded mechanism to enable easy insertion, maximize comfort and maintain reliable skin contact. The optimized earpiece shape consists of a custom scaffold, 3D printed using Tough 1500 resin (Formlabs, USA), a material compliant with ISO 10993-1 standards for prolonged skin contact. To accommodate anatomical variability, three size options were produced (range of heights: 25–29 mm (S), 28–32 mm (M), and 31–35 mm (L)). Embedded in the scaffold, the electrodes were composed of two 9x8mm folded 925 sterling silver components, designed to contact the anterior surfaces of the cymba concha and cavum concha. The total weight of the device is 158.4 grams (stimulator unit: 130 grams, earpiece: 5.5 grams, motion-tracker: 22.9 grams).

### taVNS parameter settings and stimulation logic

The SmartVNS system delivers taVNS via electrodes placed on the contralateral ear relative to the trained arm. Prior to electrode placement, the electrode are prepared using Signa Spray (Parker Laboratories, USA) to ensure optimal conductivity and reduce impedance with the skin. Stimulation was delivered as a constant current using a square biphasic waveform. The pulse width was fixed at $250 \mu$s ($250 \mu$s positive phase, $100 \mu$s inter-phase gap, $250 \mu$s negative phase) and the frequency was set at 25 Hz [17, 22, 24]. Stimulation intensity was individualized: participants progressively adjusted the current prior to each session to reach their personal sub-pain threshold by pressing the plus and minus buttons of the stimulator unit [22, 24]. The maximum output of the device was limited to $2500 \mu A$. No burst mode or explicit duty cycle was employed; instead, stimulation was continuously applied for 1 s in response to a detected movement, without a predefined off-period. After each 1-second stimulation, the stimulation logic required a period of rest (no movement, as predicted by the algorithm) before a new movement could be detected and a new stimulation applied, thereby ensuring that stimulation was not applied continuously.

The movement detection logic is based on sensor rotation in space, defined by the unit vector along the axis from the elbow to the fingers. Angular velocity, calculated using a 9-axis VQF filter [29] over 200 ms windows (24 IMU samples at 120Hz) every 100 ms, is normalized by a maximum rate ($\pi /2$ rad/s). To determine a movement event, this normalized velocity is smoothed by averaging the last three estimations. To accommodate diverse abilities and rehabilitation exercises, a dynamic threshold was implemented [30]. Parameters of this algorithm were tuned based on preliminary pilot data acquired during standard therapy. Threshold is continuously computed as the 50th percentile of predictions from the last 10 s, bounded between 30% and 60% of the maximum normalized speed.

### Study overview

To assess the feasibility, usability and performance of a novel closed-loop movement-paired taVNS approach, a four-week study was carried out (Fig. 2). Patients were recruited from the cereneo clinic, Center for Neurology and Rehabilitation (Weggis, Switzerland) and gave written informed consent in accordance with the Declaration of Helsinki.

The study population comprised individuals aged 18 years and above with confirmed residual motor impairments resulting from a prior stroke, traumatic brain injury, or SCI. Enrollment required participants to be a minimum of 1 week post-injury. Individuals were excluded from participation if they exhibited untreated major depression, significant cognitive and/or communication impairments, or comprehension and/or memory deficits that could potentially compromise informed consent, adherence to therapy protocols, or the accurate reporting of adverse events. Further exclusion criteria included a history of neurological conditions such as epilepsy, current involvement in any other research trial, pregnancy, the presence of a pacemaker or any other implanted electrical device, and the current use of any medication or undergoing any procedure known to interfere with vagus nerve function. Final inclusion in the study was contingent upon successful completion of a clinical screening performed by healthcare professionals, confirming adherence to all eligibility criteria.

Participants followed standard of care personalized upper limb therapy while wearing the SmartVNS device, 40 min a day, five times a week, for a total of 20 sessions. Therapists were instructed to perform standard exercises, while privileging those involving movements at the wrist, where the sensor is located.

The setup process, performed by the patient, (if no severe bilateral upper-limb impairments) was timed at every session. It involved four main steps all performed with the unaffected arm: (1) securing the IMU sensor on the affected arm (wrist level), (2) positioning the neck-worn stimulator, (3) inserting the ear electrode and 4) calibrating the stimulation intensity with plus and minus buttons.

Additionally, in the first session, at mid-intervention (session 10) and in the last session, therapy was recorded using a camera, and therapists were instructed to continuously press a manual button during movements of the patient. This was done in order to validate the movement detection algorithm and to compare it to the conventionally-used method of manual triggering [10, 31]. Movement data was analyzed on day 1 and day 20 of the intervention period. Furthermore, patients and therapists answered the Usability Metric for User Experience (UMUX) [32] questionnaire to assess the functionality and usability of the SmartVNS system. Adverse events or discomfort related to SmartVNS were monitored and documented.

Finally, clinical routines assessments (including Fugl Meyer Assessment-Upper Extremity (FMA-UE) [33] or the International Standards for Neurological Classification of Spinal Cord Injury (ISNCSCI; ASIA motor subscore) [34], Action Research Arm Test (ARAT) score [35], Box and Block Test (BBT) [36]) were collected before and after the study. An ARAT score of 0 was assigned to participants who were unable to perform the test as a result of profound motor impairments.

The study protocol was approved by the local cantonal medical and ethical committee (BASEC ID: 2024-01425, ClinicalTrials.gov: NCT06623721).

### Data analysis

For the purpose of evaluating the performance of the automatic movement detection algorithm, video recordings of therapy sessions were used to establish a ground truth of movement events. A movement event was defined as a continuous sequence of at least 200 ms (or 5 frames in a video of 24 frames per second), where discernible changes in the position or/and orientation of the target wrist of the subject could be observed. Conversely, a rest event was defined as a continuous sequence of at least 200ms (5 frames) where no such discernible changes were observed. Trained researchers labeled the videos with “movement" or “rest" labels, being blinded to when therapists where defining movements or when the patients were receiving stimulation. Frames with visual occlusion of the wrist were excluded from analysis, as were movements with a duration outside the range of [0.2s, 30 s].

To assess the system’s performance in distinguishing between movement and rest, we employed two standard metrics: precision and sensitivity. Precision quantifies the proportion of stimulations happening during movement:

1 $$\begin{aligned} \text {Precision} = \frac{\text {True Positives (TP)}}{\text {True Positives (TP)} + \text {False Positives (FP)}} \end{aligned}$$

A high precision value indicates a low rate of false positives (i.e., minimizing stimulations during rest periods). Sensitivity measures the proportion of actual movement events that were successfully stimulated:

2 $$\begin{aligned} \text {Sensitivity} = \frac{\text {True Positives (TP)}}{\text {True Positives (TP)} + \text {False Negatives (FN)}} \end{aligned}$$

A high sensitivity value indicates a low rate of false negatives (i.e., the system successfully detects and stimulate a large proportion of all true movements). Together, precision and sensitivity provide complementary insights: precision emphasizes the accuracy of predictions, while sensitivity emphasizes the completeness of detection.

The objective of the stimulation logic was to pair functional upper-limb movements with a precisely timed stimulation event, as done with therapist manually trigerring stimulation in invasive VNS [10, 31]. To evaluate the performance of the entire stimulation logic, we used our precision and sensitivity metrics in two different, complementary settings: one focused on stimulation events (*event-based*), and a second on movement detection (*point-wise*). This distinction is crucial, as not all movement predictions from IMU resulted in a stimulation, due to our specific stimulation logic. In the event-based analysis, we considered stimulation triggered automatically by the SmartVNS system (event: 1 s stimulation) and by the therapist (event: phantom 1 s stimulation, starting when therapist starts pressing the trigger), and compared them to the video labeled ground truth. The term phantom stimulation refers to therapist-triggered events that do not actually induce stimulation but are logged for post-hoc analysis. For the purpose of these calculations, a stimulation event was classified as a TP if at least 0.3 s of its 1 s duration occurred during a labeled movement. The point-wise analysis complemented this by assessing the ability to detect movement at every data timestamp, regardless of whether a stimulation was triggered. Here, we compared the algorithm’s real-time movement prediction (IMU-based algorithm, 10% normalized speed threshold) with the therapist’s continuous, button-press recording (not only when starting to press). Together, these two analyses provide a robust evaluation of both the temporal precision of the stimulation delivery and the underlying accuracy of the movement detection algorithm.

Furthermore, we extracted for each session the number of stimulations received, calibration time and average stimulation intensity from the device log files. Since patients could adjust the intensity after therapy began, all intensity changes were factored into the total calibration time. The average stimulation intensity was calculated as a time-weighted average to accurately reflect the patient’s stimulation experience throughout the session, rather than just the final intensity.

Statistics were performed using Prism 10.4.1. Comparison of stimulation and algorithm precision and sensitivity of SmartVNS versus therapist phantom stimulation analysis were performed using paired-t tests. Data reported are mean ± SD unless otherwise specified, p-values were considered significant at p < 0.05.

## Results

Initially, eleven patients were recruited for this study; however, two withdrew their participation for medical reasons unrelated to the research. Consequently, a total of nine patients (n= 6 stroke, n=3 SCI) completed the study. Additionally, one participant (P08) completed 12 of the 20 planned sessions due to an early discharge from the clinic. Further demographic and clinical characteristics of the participants are presented in Table 1. To assess the feasibility and generalizability of the SmartVNS system, we included a heterogeneous group of participants with upper limb motor impairments, ranging from very severe (ARAT = 0 points) to mild impairments (ARAT = 52 points). Baseline measurements for the stroke patients showed an FMA-UE score of 21.8 ± 14.3. For all patients, the ARAT score was 21.1 ± 19.9 and the Box and Block Test score was 16.0 ± 20.2. All therapy sessions were conducted by trained physical therapists who followed their standard rehabilitation protocols. The integration of SmartVNS was designed to be as minimally disruptive as possible, and therapists were instructed to deliver their usual therapy routines while incorporating SmartVNS as an adjunct.

**Table 1:** Participant characteristics

ID	Diagnosis	Etiology	Age (y)	Sex	Time Since Injury (mo)	Trained Arm	Baseline ARAT
P01	Stroke	Ischemic	37	F	31	Right	6
P02	SCI	Traumatic	41	M	3	Right	4
P03	SCI	Traumatic	67	M	10	Right	27
P05	Stroke	Ischemic	76	F	1	Left	47
P06	Stroke	Hemorrhagic	72	F	33	Left	0
P07	Stroke	Ischemic	72	M	3	Left	0
P08	Stroke	Ischemic	72	M	4	Right	31
P09	Stroke	Hemorrhagic	61	F	22	Right	23
P11	SCI	Traumatic	43	M	7	Right	52
Total	Stroke: 6 SCI: 3	–	60 ± 15	F: 4 M: 5	13 ± 12	Right: 6 Left: 3	21 ± 19

Values are shown for each participant; summary statistics are presented in the final row. Continuous variables are mean ± SD; categorical variables are counts

ARAT: Action Research Arm Test

### Hemiparetic patients are able to setup the SmartVNS system themselves

At the start of each session, participants were instructed to self-administer the SmartVNS system, unless they suffered from severe bilateral impairments, where therapists performed the set up (n = 2). On average patients required 31 ± 20 s (n = 7) to complete setup. Following placement, participants were instructed to gradually increase the stimulation intensity reaching their individualized threshold (934 $\pm$ 255 $\mu$A, on average 51.9 ± 12.3 s to complete, n = 9). An example of the complete setup done independently by a stroke patient during the first session of therapy is shown in Fig. 3.

### SmartVNS provides consistent stimulation across diverse patient impairments

**Fig. 4:**
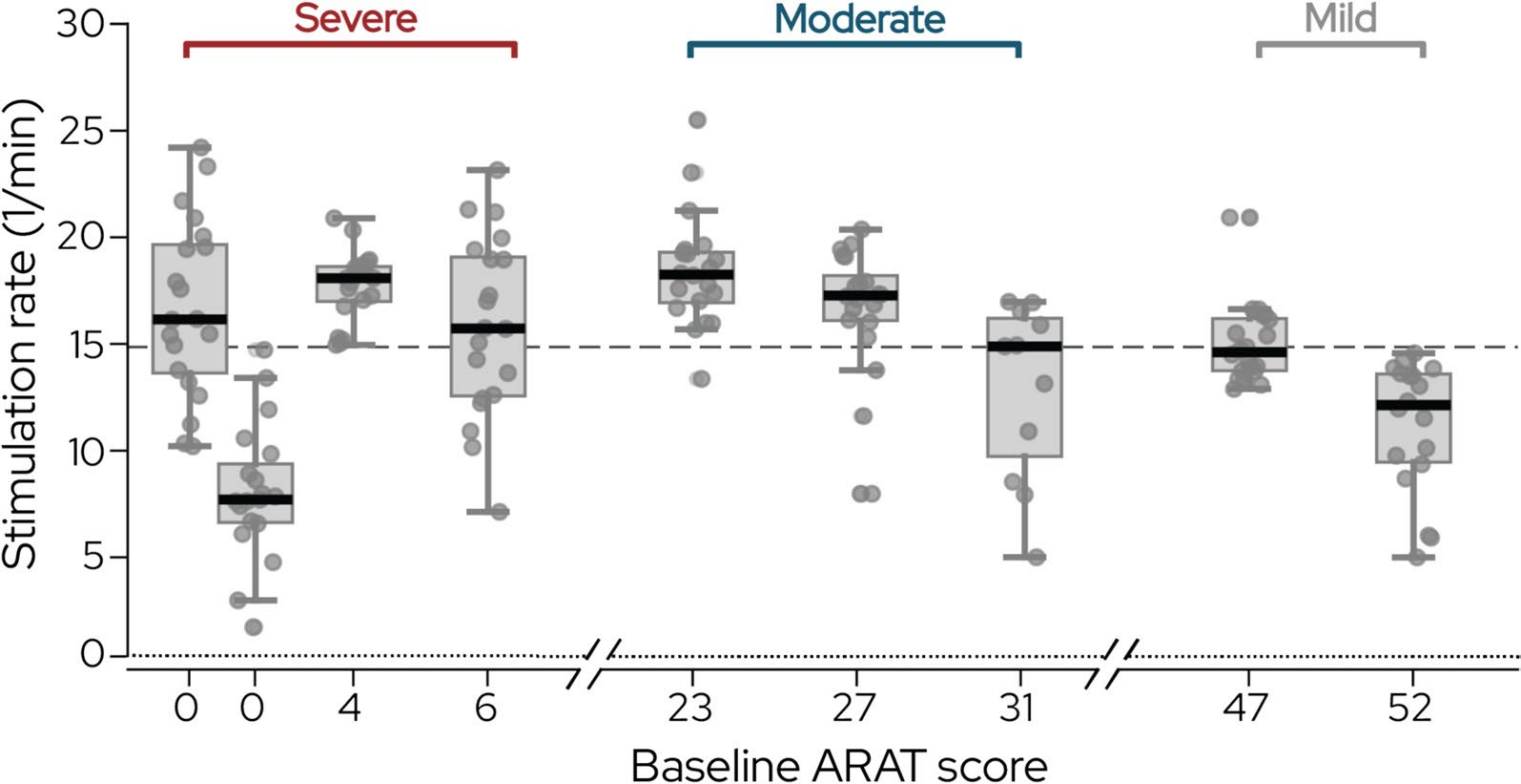
Stimulation rate versus subject baseline ARAT score. Each boxplot represents a single patient (n = 9), with individual data points corresponding to the average stimulation rate recorded during a single therapy session. Dashed line indicates average of 14.7 stimulations/minute across patients. Stimulation logic provided similar range of stimulation dosage among patients with different abilities (baseline ARAT scores) and performing different type of exercises

All patients in our study, regardless of their specific impairments or the type of exercises they performed, were able to effectively use the system. To evaluate the system in real-world clinical conditions, we examined the performance of the SmartVNS stimulation algorithm. Given the heterogeneity of the participant population, we first assessed the robustness of the stimulation logic used for movement-triggered stimulation. As shown in Fig. 4, the system achieved a consistent stimulation rate between sessions and participants, with an average of 14.7 stimulations/min (n = 9), and low variability between participants with a standard deviation of 3.4 stimulations/min (n = 9). By comparison, therapists manually triggered stimulations at a rate of 5.7 ± 4.5 phantom stimulations per minute.

### SmartVNS has equivalent precision to manual tracking with higher sensitivity

**Fig. 5:**
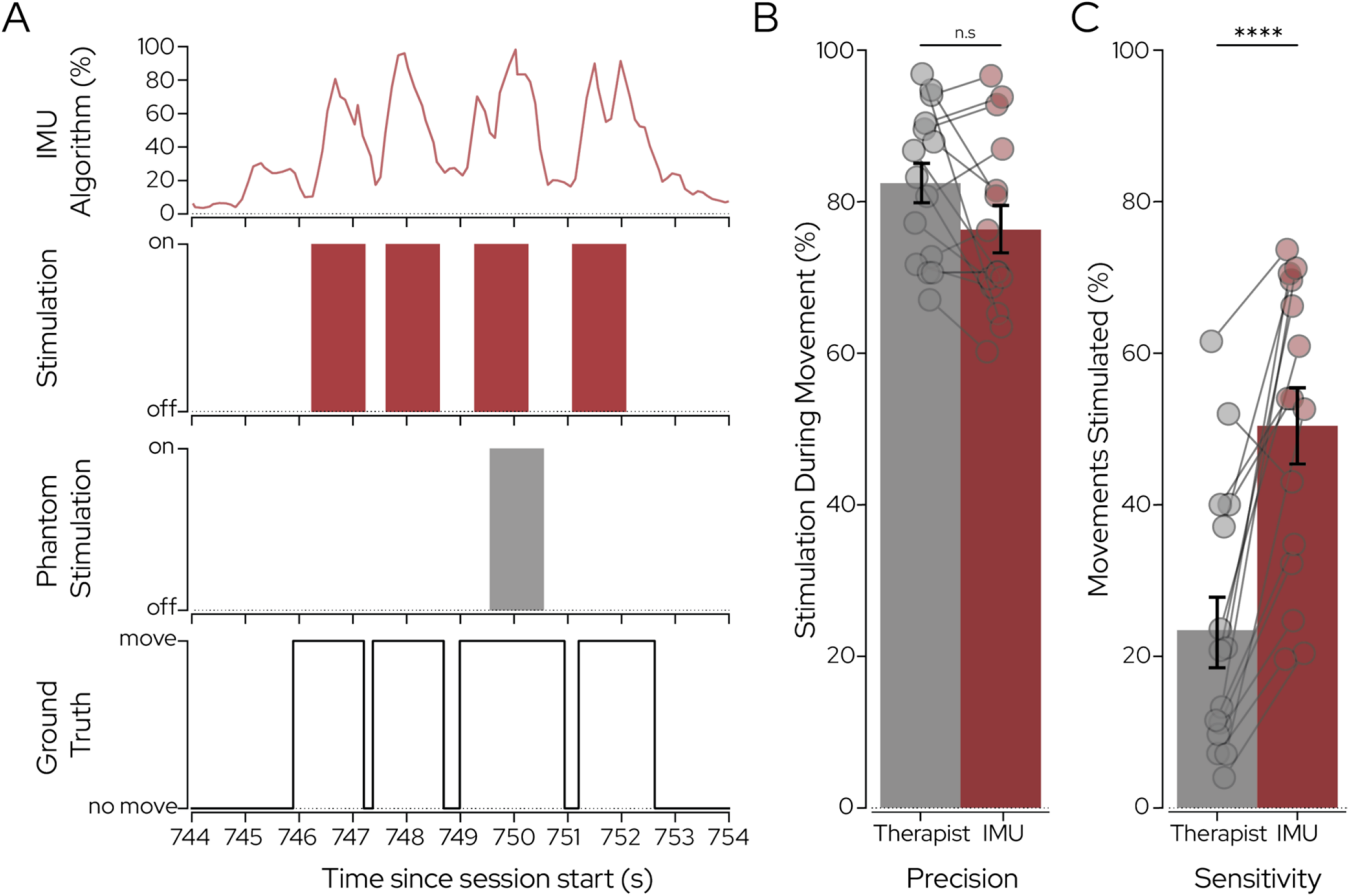
Stimulation logic performance: Event-based analysis (n = 15 sessions). Two triggering methods, therapist manual phantom triggering (grey) and IMU-based automated stimulation (red), are compared against video recordings (ground truth, black). **A** Example snippet of raw sensor data (top panel), 1 s stimulation events (second panel), 1 s therapist phantom stimulation events (third panel), and ground truth labeling (bottom panel). **B** Precision measures the proportion of stimulations during movements, while **C** Sensitivity measures the proportion of movements successfully stimulated. Individual points denote the average performance per session, and lines connect data from the same session to compare therapist versus IMU performance. Data shown are mean ± SEM; n.s.=p > 0.05, ****=p < 0.0001

To further validate movement detection accuracy, we compared automated stimulation timings with therapist-initiated button presses during goal-directed movements, in the event-based context. Video annotations provided ground truth onset and offset times. Data from three sessions were excluded from the analysis: one due to a computer communication error, one due to missing therapist labels, and one due to the subject leaving the clinic early. There was no significant difference between stimulation of movements detected by SmartVNS (76.3 ± 3.1%, mean ± SEM) compared to therapist-manual phantom stimulation (82.4 ± 2.6%, mean ± SEM); (Fig. 5B, paired t-test t(14) = 2.057, p = 0.059). Furthermore, SmartVNS stimulated a significantly greater proportion of movements (50.4 ± 5.0%, mean ± SEM) compared to therapist phantom stimulation (23.4 ± 4.7%, mean ± SEM); (Fig. 5C, paired t-test t(14) = 5.617, p < 0.0001).

**Fig. 6:**
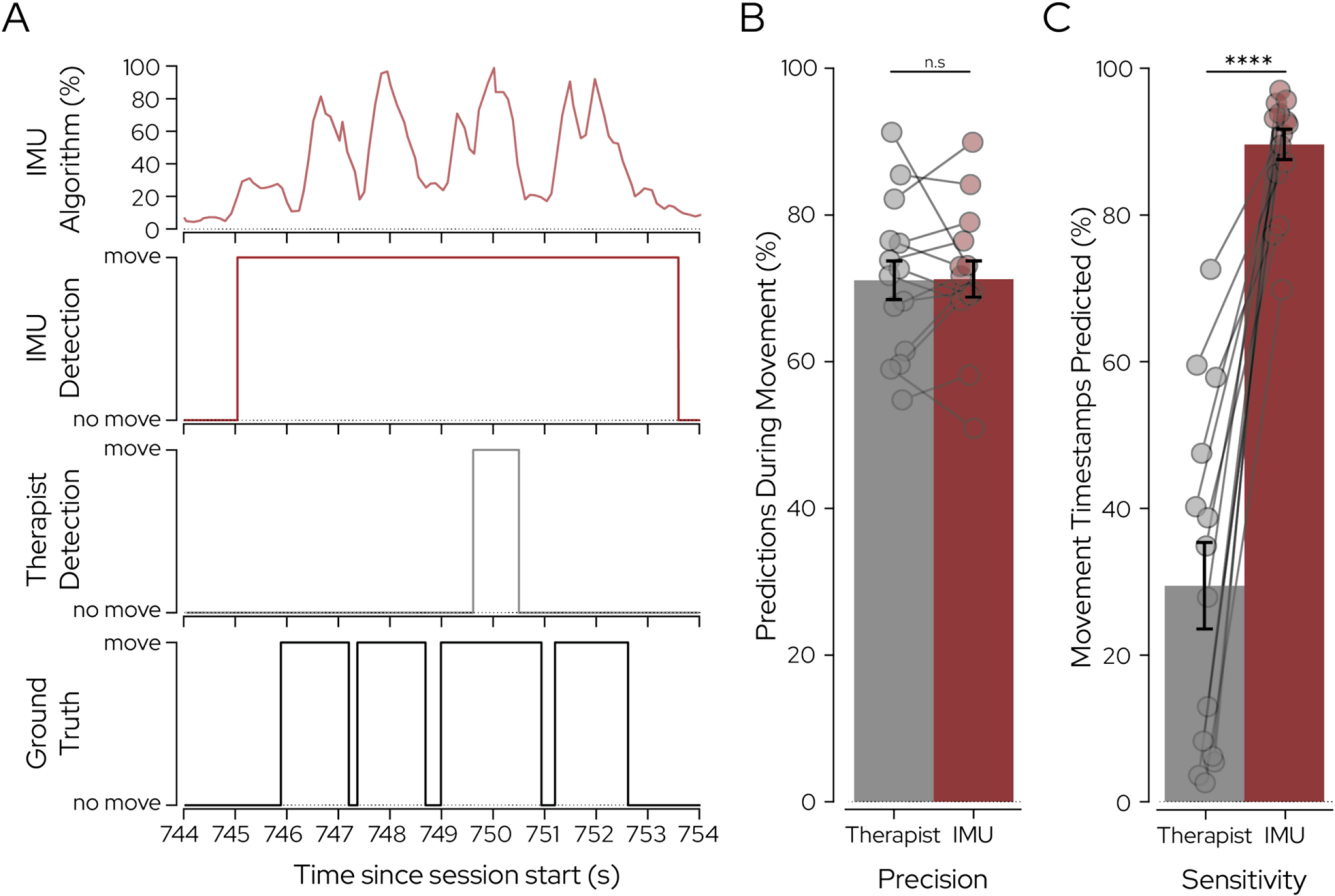
Movement detection performance: Point-wise analysis (n = 15 sessions). Two labeling methods, therapist pressing a manual button (grey) and IMU-based automated detection (red), are compared against video recordings (ground truth, black). The IMU algorithm used a 10% threshold. **A**) Example snipped of raw sensor data (top panel), point-wise IMU detection (second panel), point-wise therapist phantom stimulation (third panel), and ground truth labeling (bottom panel). **B** Point-wise precision, defined as the proportion of predicted timestamps that occurred during movements. **C** Point-wise sensitivity, defined as the proportion of movement timestamps that were successfully detected. Data shown are mean ± SEM; n.s.=p>0.05, ****= p<0.0001

The SmartVNS stimulation logic is designed to avoid continuous stimulation. Following the delivery of a 1-second burst, a new stimulation is only made eligible once the movement signal has fallen below the adaptive threshold. To investigate the system’s capability to detect movement, and eventually support alternative stimulation logics (e.g., continuously stimulating when movement is detected), we evaluated the performance of the movement detection algorithm without applying the stimulation logic. Point-wise analysis showed that the algorithm, using a 10% normalized speed threshold, was as precise as therapist manual labeling (Fig. 6B; paired t-test: t(14) = 0.073, p = 0.943). On average, it detected 89.1% of movement timestamps, compared to 29.4% detected by therapists (Fig. 6C; paired t-test: t(14) = 9.261, p < 0.0001).

### SmartVNS is highly accepted by patients and therapists

**Fig. 7:**
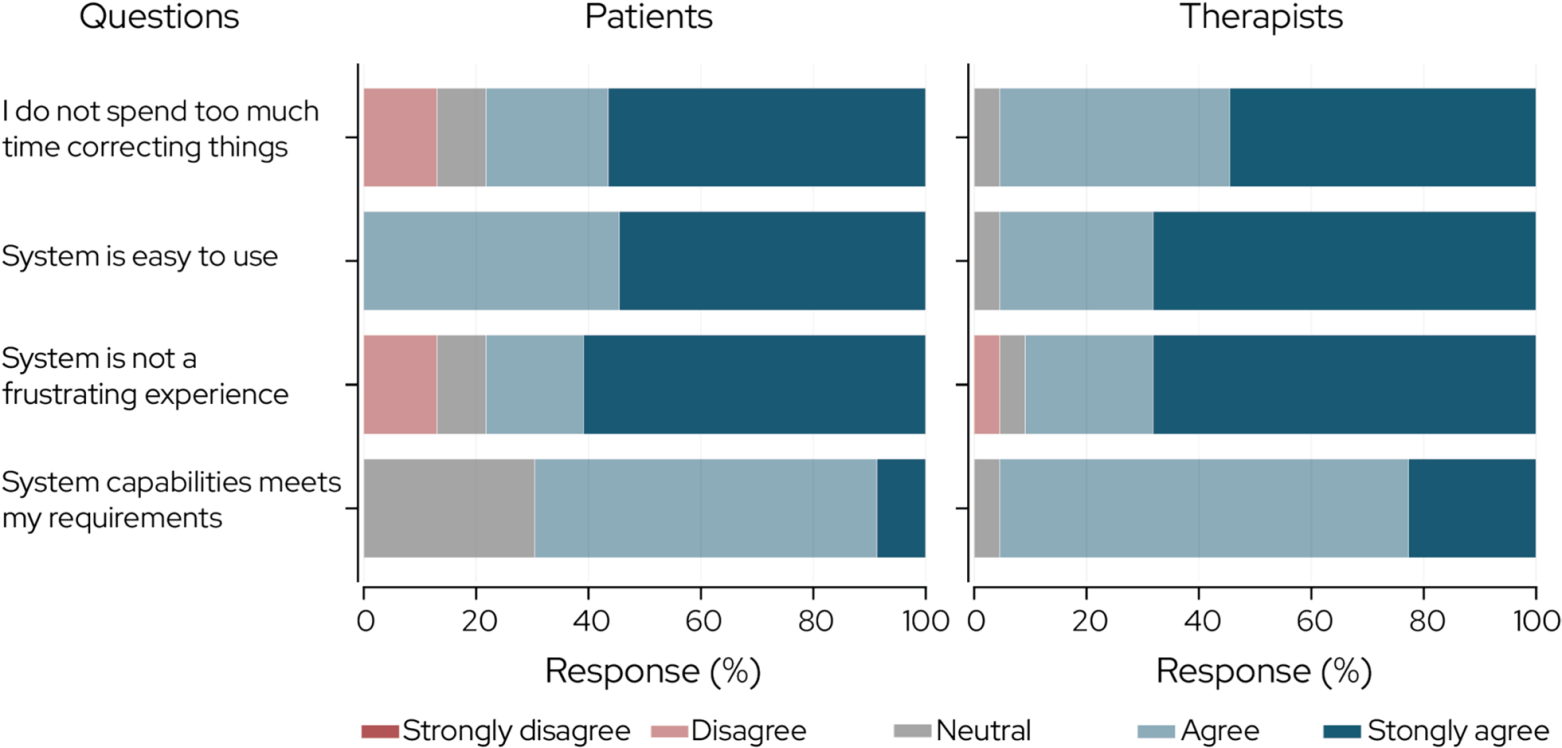
Likert scale visualization of UMUX score for patients (n = 8, 23 total response) and therapists (n = 8, 22 total responses), combining all sessions

To evaluate the acceptability and usability of SmartVNS, we administered a standardized questionnaire, the UMUX [32], on session 1, 10 and 20 of the study to both patients and their therapists for those sessions. The assessment aimed to capture ease of handling, system usability, and user satisfaction. Due to incomplete data, one participant was excluded from this analysis, and another participant’s UMUX scores are only available for session 1 and 10 because the individual left the clinic early. The general usability among the combined patients and the therapists was highly rated, as shown in Fig. 7, with an average UMUX score of 85 ± 12%, indicating excellent usability. Among patients, the average UMUX score was 82 ± 12% (n = 8, 23 total responses, Supplementary Materials, Fig. A1 and Table A1) and therapists rated the system slightly more favorably at 89% ± 12% ( n = 8, 22 total responses, Supplementary Materials, Table A2).

Importantly, no serious adverse events were reported during the study. Minor adverse events were limited to three cases of transient headache.

### Preliminary evidence that the SmartVNS-adjunct therapy lead to improvements in both stroke and SCI populations

As a secondary outcomes of the study, we collected clinical outcome measures at the beginning of the study (session 1) and after the intervention (session 20). Detailed assessments of ARAT, BBT and FMA-UE (stroke subgroup only) are presented in Supplementary Materials, Table A3. One participant was excluded from this analysis as the individual declined to complete post-intervention assessments. Two participants were not assessed for ARAT and BBT for the intervention due to too severe impairments to realize the assesment, and a score of 0 was given for both assessments and included in the average.

**Fig. 8:**
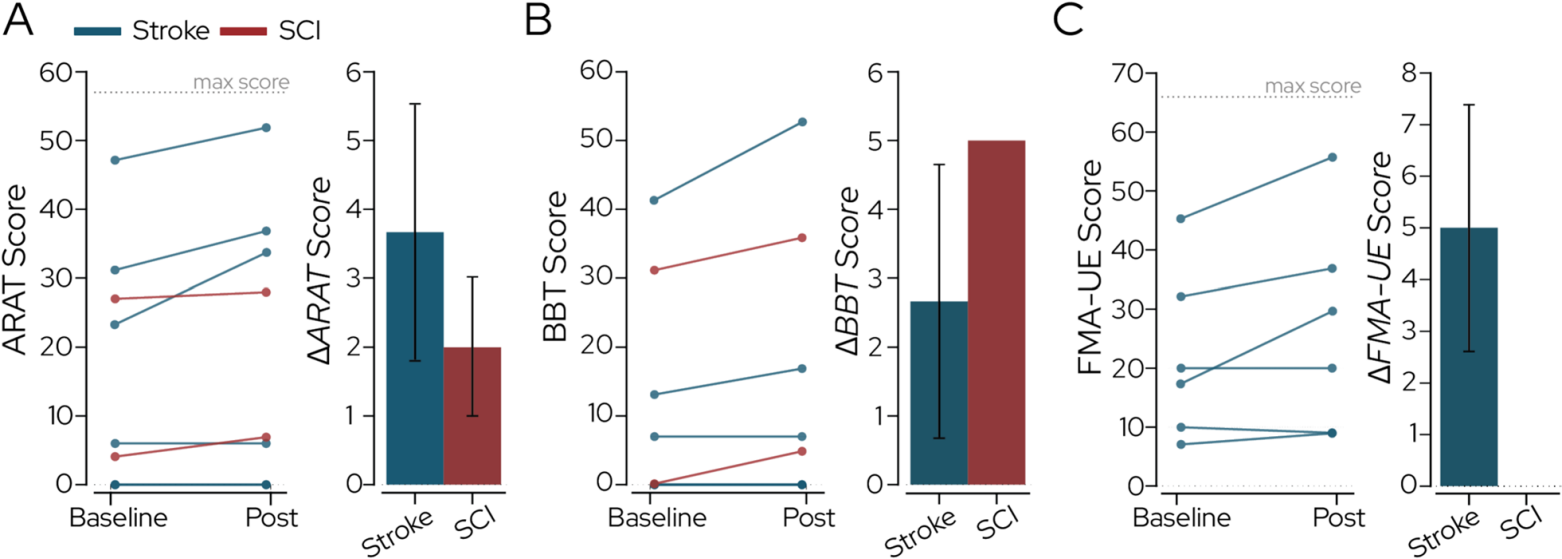
Assessments pre-post intervention. **A**) Pre-post scores from ARAT with average change of assessment scores across the intervention in right sub-panel; **B**) same as in A for BBT, and **C**) same as in A for FMA-UE for the stroke population. Blue colors indicate stroke patients, red colors indicate SCI patients. Data shown are mean ± SEM

Overall, the results show modest but consistent improvements in all three outcome measures (Fig. 8, see Appendix Table A3 for more details). ARAT scores improved from baseline with a mean change of 3.3 ± 1.4 points (mean ± SEM, n = 8; range: 0–11). BBT scores similarly showed a mean increase of 3.3 ± 1.5 blocks (mean ± SEM, n = 8; range: 0–12). In the stroke subgroup, FMA-UE scores show a mean improvement of 5.0 ± 2.4 point (mean ± SEM, n=6; range: -1–3).

## Discussion

We developed the SmartVNS system, a fully wearable, closed-loop, movement-paired taVNS stimulation device designed for use in neurorehabilitation of the upper limb. Our results demonstrate that SmartVNS is feasible to use in clinical settings for a wide range of upper limb deficits, as demonstrated by: (i) patient’s ability to set up and calibrate the device themselves or with minimal supervision from therapists; (ii) its easy integration within standard rehabilitation exercises; (iii) accurate stimulation delivery during upper limb movements in patients with severe to mild impairment; and (iv) high usability scores.

### Automated taVNS is feasible in clinical settings

A common barrier to the adoption of neurorehabilitation technologies, specifically neuromodulation approaches, is poor usability. Many systems require complex and time-consuming setup procedures, calibration, or sensor placement that can be challenging for both patients and therapists to manage independently. This not only limits their practical use in the real world, but also increases reliance on specialized training [37]. Furthermore, for patients with motor or cognitive impairments, the complexity of these systems can further reduce engagement and adherence. In addition to usability challenges, many neurotechnologies introduce additional workflow demands for therapists or caregivers. These include manual stimulation triggers or adapting therapy protocols to fit the intended use of the device. Without integration into existing clinical workflows, these added responsibilities can reduce overall efficiency, increase cognitive load, or create resistance to adoption, especially in clinical settings where time and attention are limited [38, 39].

Our findings suggest that SmartVNS overcomes many of these usability challenges, even in patients with severe impairments (ARAT=0, n = 2). At the beginning of each session, patients were instructed to independently setup SmartVNS unless they had significant bilateral upper limb impairments (n = 2, ARAT = 4 and ARAT = 27), in which case the physical therapist assisted. On average, the complete setup and stimulation calibration process took less than 2 min, which is significantly faster than other neuromodulation techniques such as repetitive transcranial magnetic stimulation (rTMS) or other methodologies of movement-gated taVNS that require placement of electrode patches over different anatomical landmarks, demanding external supervision. Importantly, once the setup was complete, the therapists delivered the same therapy as they would to their patients. The presence of the device did not require any modification to therapeutic activities and did not interfere with patient-therapist interactions. Furthermore, this is reflected in the excellent usability scores of both patients and therapists. The average UMUX score was 85%, indicating strong overall satisfaction with the ease of use and integration of the system into therapy sessions. Importantly, these ratings were sustained over the three time points (session 1, 10, 20; Supplementary Materials, Fig. A1 and Table A1), suggesting that the system maintains usability over time and is not prone to novelty bias or frustration for patients. Therapists rated the system highly, underscoring its compatibility with clinical routines and minimal additional burden during sessions (Table A2).

### SmartVNS was able to deliver precise and consistent stimulation to a wide range of motor impairments

In this work, we introduced and evaluated a novel method for controlling taVNS delivery in response to functional upper-limb movements, using an IMU for automatic detection and closed-loop stimulation. The adaptive stimulation logic was specifically designed to prevent the stimulation of involuntary short movements (e.g., muscle twitches, tremor etc.) and avoid over-stimulation during continuous movements. This design choice proved effective, with the system demonstrating a high precision and sensitivity (Fig. 5). Notably, the automated system stimulated more than double the number of movements compared to manual triggering via a therapist (50.4% sensitivity vs. 23.4% sensitivity) while maintaining comparable precision. Given the diverse abilities of the patient cohort, these findings suggest that the implemented stimulation logic is both sensitive and adaptable to individual movement profiles. This adaptability was further demonstrated by the highly consistent stimulation rate across patients (Fig. 4), with a low inter-patient deviation, potentially attributable to the use of a dynamic threshold that normalizes detection across varying movement velocities, thereby supporting its applicability in diverse rehabilitation populations.

In contrast to conventional invasive VNS protocols, which typically deliver 5 stimulations per minute in stroke patients [10], and recent efforts to achieve similar stimulation rates autonomously using wearable sensors in SCI populations [11, 30], our system delivered an average of 15 stimulations per minute. The varying effects of VNS doses present a key consideration for clinical application in neurorehabilitation. Although an increase in stimulation dose is hypothesized to improve synaptic plasticity, evidence from animal studies indicates that further increases in dose do not yield additional recovery benefits [40].

### Challenges of therapist manually-triggered stimulation

While therapists triggered stimulation at rates comparable to those reported in previous studies [10, 11, 30], our results demonstrated that our IMU-based stimulation was more sensitive than phantom stimulation (i.e., more movements were stimulated). We identified two key factors that may explain this discrepancy: i) how therapists interpret goal-directed movement, and ii) the physical and cognitive demands already placed on therapists during rehabilitation.

First, many rehabilitation exercises consist of two distinct phases of movement. For example, in a cup drinking task, a series of coordinated arm extension (reaching forwards), grasping, lifting and controlled flexion to the mouth are required. Therapists were instructed to trigger phantom stimulation during phases of whole arm movements (extension and flexion phases). However, we observed that phantom stimulation was often applied continuously, as each phase was deemed important (e.g., including the grasping phase). This suggests that therapists instinctively prioritize delivering stimulation for the entire repetition, pressing the button continuously throughout the movement rather than pressing twice to separately mark each phase.

In contrast, our algorithm was agnostic to movement type and consistently triggered stimulation during both the extension and retraction phases. Furthermore, while our current stimulation logic was designed to mimic manual stimulation during movement initiation, future studies could explore alternative approaches. Animal models have demonstrated that pairing VNS with outcome-driven events (e.g., successful retrieval of a pellet) can improve motor learning, a key component for stroke recovery [41–43]. Future work studies could further refine stimulation protocols to reinforce outcome or quality-related movements to possibly enhance recovery outcomes.

Second, and perhaps more significant, therapists often provide physical assistance to patients, especially patients with lower abilities, such as supporting the arm or body or handling different therapeutic tools. In many cases, this limited their ability to trigger the phantom stimulation, as they cannot operate the button while simultaneously assisting the patient.

While manual triggering is a useful research tool, its practical implementation in clinical settings is hindered by many challenges encountered in this study. Therefore, the development of automatic assessment approaches seems essential for the effective translation and scalability of these interventions into clinical practice.

### SmartVNS may provide additional clinical benefit

VNS has emerged as a promising neuromodulation technique to support upper-limb neurorehabilitation. Multiple recent studies have shown encouraging results using both movement-gated implanted and non-invasive forms of VNS in stroke survivors, with a particular emphasis on moderate to mild impairments. For example, the VNS-REHAB trial [10] recruited participants with FMA-UE scores between 20 and 50, with a mean baseline of 34.4 ± 8.2 points. Badran et al. [21] included individuals with FMA-UE $\le$ 58 and a minimum of 3$\circ$ of active wrist flexion, reporting a baseline score of 36.56 ± 7.94. Redgrave et al. [22] had baseline ARAT impairments of 21.3 ± 25.2, also consistent with moderate impairments.

In contrast, our cohort included participants with a wider range of motor impairments, including more severely affected individuals, which was the objective in this pilot study focusing on feasibility and not efficacy. At baseline, the average ARAT score in our cohort was 17.25 ± 17.31 (range: 0 to 52), and the average FMA-UE score (stroke participants only) was 21.83 ± 14.33 (range: 7 to 45), well below the average of the above-mentioned studies. The observed FMA-UE improvements in the stroke subgroup (stroke-only: 5.0 ± 2.4 SEM) falls within the range of changes reported in iVNS (5.0 ± 4.4 SD [10]) and non-invasive taVNS trials (5.0 ± 5.00 SEM [21]).

### Limitations and future works

While this study primarily aimed to evaluate the feasibility of SmartVNS, the lack of a sham control group and the limited sample size restrict our ability to draw definitive conclusions regarding the efficacy of the intervention. The observed mean FMA-UE change of 5.0 points in the stroke subgroup approaches but does not reach the commonly reported minimal clinically important difference (MCID) threshold of 5.25 points for this scale (derived from mild-to-moderate chronic ischemic stroke populations [44]). This should be interpreted cautiously given the absence of a control arm, the heterogeneous cohort including severely impaired patients (FMA-UE < 20), and potential contribution of spontaneous recovery in subacute participants. Future studies are necessary to determine the exact efficacy of the intervention. To inform the design of these future large-scale, controlled trials, several considerations may be valuable.

Given the favorable safety profile of SmartVNS and taVNS in general, future studies should investigate its use during the acute and subacute phases of stroke and SCI. Indeed, early phase intervention may offer a critical window to maximize neuroplasticity and enhance functional recovery outcomes [45].

As the SmartVNS system demonstrated high usability in clinical settings, including independent use by patients, future work should explore the feasibility of deploying SmartVNS in home environments. This would involve validating its safety, usability, and effectiveness outside of clinical environments, potentially supporting more continuous and personalized therapy improving accessibility and promoting long-term adherence. Insights from larger ongoing controlled trials, such as TRICEPS [27] would be highly informative in guiding this direction.

## Conclusion

This study demonstrated the feasibility and usability of SmartVNS, a fully wearable, closed-loop movement-paired taVNS system designed and optimized for integration into upper-limb neurorehabilitation. The system enabled patients to set up the device themselves and robustly delivered accurate stimulation during functional movements across a wide range of impairment levels. High usability ratings from patients and therapists support its clinical integration, as the system minimized the potential added workload for therapists, helping them focus on their patients during therapy sessions. Despite a heterogeneous cohort, patients had functional improvements across the 20 sessions, which warrant for sham controlled studies to assess the efficacy of SmartVNS. These findings support SmartVNS as a scalable, user-friendly platform for neuromodulation in clinical workflows.

## Supplementary Information

Supplementary Material 1.

## Data Availability

The datasets generated and/or analyzed during the current study are available from the corresponding author upon reasonable request.

## Code availability

The code used for data processing and analysis is available from the corresponding author upon reasonable request.

## Materials availability

All custom materials and hardware used in this study are available from the corresponding author upon request.
