# Supplementary material for: Closed-loop movement-paired transcutaneous auricular vagus nerve stimulation for upper-limb rehabilitation: a feasibility study
Source: J Neuroeng Rehabil. 2026 May 20;23:220. doi: 10.1186/s12984-026-02021-7 (PMC13371290; doi:10.1186/s12984-026-02021-7)
Supplement: Supplementary file 1 — Supplementary Material 1. [file 12984_2026_2021_MOESM1_ESM.pdf]

Appendix A Supplementary Figures

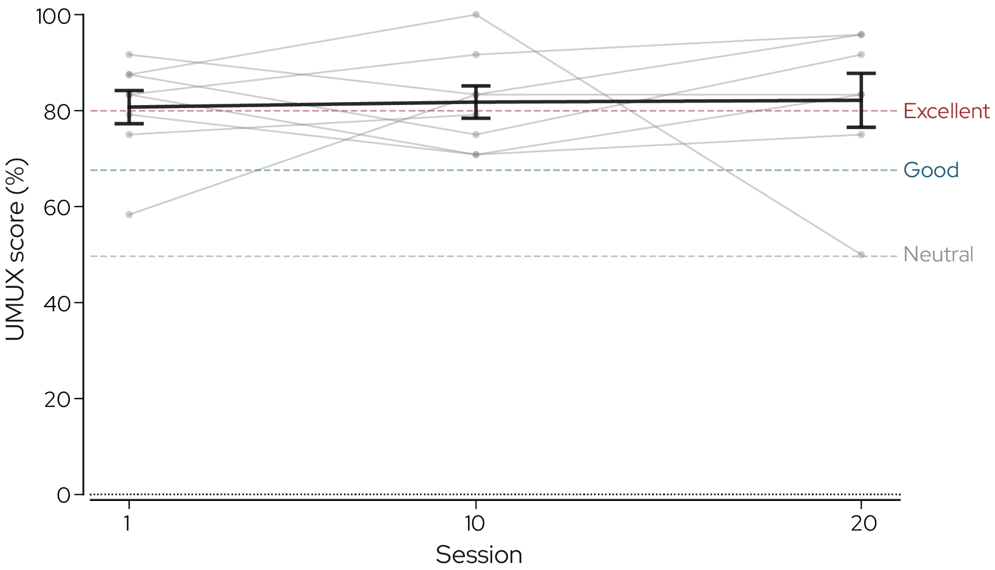

Fig. A1: UMUX evolution in patients.

Patient	Session #1	Session #10	Session #20
P01	79.17	70.83	75.00
P02	87.50	75.00	91.67
P03	83.33	70.83	83.33
P05	83.33	91.67	95.83
P06	91.67	83.33	95.83
P07	58.33	83.33	83.33
P08	75.00	79.17	–
P09	87.50	100.00	50.00

Table A1: UMUX scores (%) for each patient at three time points. Missing scores are denoted by "–".

Therapist	Assessment #1	#2	#3	#4	#5
T01	91	91	95	–	–
T02	66	–	–	–	–
T03	58	83	83	75	75
T04	95	100	100	100	–
T05	87	83	–	–	–
T06	83	100	95	95	95
T07	95	–	–	–	–
T08	95	–	–	–	–

Table A2: UMUX scores (%) for each therapist across different therapy sessions. Each therapist could have participated in between one and five assessments across different patients (sessions 1, 10, or 20).

Table A3: Clinical assessment scores of upper-limb function before and after intervention. "–" denotes missing information.

Subject	FMA-UE†			ARAT‡			Box & Block§		
	Pre	Post	Δ	Pre	Post	Δ	Pre	Post	Δ
P01-Stroke	20	20	0	6	6	0	0	0	0
P05-Stroke	45	56	11	47	52	5	41	53	12
P06-Stroke	7	9	2	0	0	0	0	0	0
P07-Stroke	10	9	-1	0	0	0	0	0	0
P08-Stroke	17	30	13	31	37	6	13	17	4
P09-Stroke	32	37	5	23	34	11	7	7	0
**SCI Cohort**	**ASIA Motor Score¶**			**ARAT‡**			**Box & Block§**		
	Pre	Post	Δ	Pre	Post	Δ	Pre	Post	Δ
P02-SCI	3	3	0	4	7	3	0	5	5
P03-SCI	16	–	–	27	28	1	31	36	5
P11-SCI	25	–	–	52	–	–	52	–	–

†FMA-UE MCID = 5.25 points ‡ARAT MCID = 5.7 points §Box & Block MDC 5.5 blocks ¶ASIA Motor Score MCID context-dependent
